# Redetermination of Fe_2_[BP_3_O_12_]

**DOI:** 10.1107/S1600536810029818

**Published:** 2010-08-04

**Authors:** Fei Fei Li, Hui Ju Zhang, Li Na Zhang

**Affiliations:** a Department of Physics and Chemistry, Henan Polytechnic University, Jiaozuo, Henan 454000, People’s Republic of China

## Abstract

Explorations of phases in the quaternary Fe^III^–B^III^–P^V^–O system prepared by the high temperature solution growth (HTSG) method led to single-crystal growth of anhydrous diiron(III) borotriphosphate, Fe_2_[BP_3_O_12_]. This phase has been synthesized previously as a microcrystalline material and its structure refined in space group *P*3 from powder X-ray diffraction data using the Rietveld method [Chen *et al.* (2004 ▶). *J. Inorg. Mater.* **19**, 429-432]. In the current single-crystal study, it was shown that the correct space group is *P*6_3_/*m*. The three-dimensional structure of the title compound is built up from FeO_6_ octa­hedra (3.. symmetry), trigonal–planar BO_3_ groups (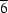 symmetry) and PO_4_ tetra­hedra (*m*.. symmetry). Two FeO_6_ octa­hedra form Fe_2_O_9_ dimers *via* face-sharing, while the anionic BO_3_ and PO_4_ groups are connected *via* corner-sharing to build up the [BP_3_O_12_]^6−^ anion. Both units are inter­connected *via* corner-sharing.

## Related literature

Reviews on the crystal chemistry of borophosphates were given by Kniep *et al.* (1998 ▶) and Ewald *et al.* (2007 ▶). For the previous powder study of Fe_2_[BP_3_O_12_], see: Chen *et al.* (2004 ▶). For the structure of a related borophosphate, see: Zhao *et al.* (2009 ▶). Meisel *et al.* (2004 ▶) have reported the structure of V_2_[BP_3_O_12_] and Mi *et al.* (2000 ▶) that of Cr_2_[BP_3_O_12_].

## Experimental

### 

#### Crystal data

Fe_2_[BP_3_O_12_]
*M* *_r_* = 407.42
Hexagonal, 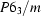

*a* = 8.0347 (8) Å
*c* = 7.4163 (13) Å
*V* = 414.63 (9) Å^3^
*Z* = 2
Mo *K*α radiation
μ = 4.15 mm^−1^
*T* = 293 K
0.15 × 0.05 × 0.05 mm

#### Data collection

Rigaku Mercury70 CCD diffractometer
Absorption correction: multi-scan (*ABSCOR*; Higashi, 1995 ▶) *T*_min_ = 0.575, *T*_max_ = 0.819
3247 measured reflections
345 independent reflections
338 reflections with *I* > 2σ(*I*)
*R*_int_ = 0.042

#### Refinement

*R*[*F* ^2^ > 2σ(*F* ^2^)] = 0.035
*wR*(*F* ^2^) = 0.072
*S* = 1.07
345 reflections
33 parameters
Δρ_max_ = 0.58 e Å^−3^
Δρ_min_ = −0.78 e Å^−3^

### 

Data collection: *CrystalClear* (Rigaku, 2004 ▶); cell refinement: *CrystalClear*; data reduction: *CrystalClear*; program(s) used to solve structure: *SHELXS97* (Sheldrick, 2008 ▶); program(s) used to refine structure: *SHELXL97* (Sheldrick, 2008 ▶); molecular graphics: *DIAMOND* (Brandenburg, 2004 ▶); software used to prepare material for publication: *SHELXTL* (Sheldrick, 2008 ▶) and *PLATON* (Spek, 2009 ▶).

## Supplementary Material

Crystal structure: contains datablocks I, global. DOI: 10.1107/S1600536810029818/wm2377sup1.cif

Structure factors: contains datablocks I. DOI: 10.1107/S1600536810029818/wm2377Isup2.hkl

Additional supplementary materials:  crystallographic information; 3D view; checkCIF report

## Figures and Tables

**Table 1:** Selected bond lengths (Å)

Fe1—O3^i^	1.929 (2)
Fe1—O2	2.103 (2)
P1—O3	1.507 (3)
P1—O2	1.538 (3)
P1—O1	1.586 (3)
O1—B1	1.357 (3)

Symmetry code: (i) 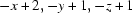.

**Fig. 1.**
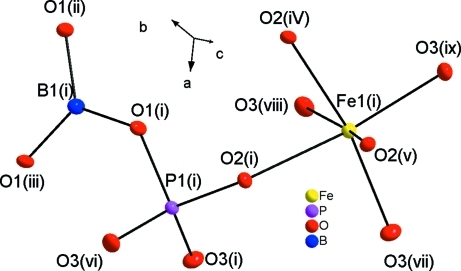
Section of the structure of Fe2[BP3012] with the atom labelling scheme. The displacement ellipsoids are drawn at the 50% probability level. [Symmetry codes: (i) 2 - x, 1 - y, 1 - z; (ii) x-y, -1 + x, 1 - z; (iii) y, 1 - x + y, 1 - z; (iv) 1 - x + y, 1 - x, 0.5 - z; (v) 1 - y, x-y, z; (vi) x, y, 0.5 - z; (vii) 2 - y, 1 + x-y, z; (viii) 1 - x + y, 2 - x, 0.5 - z.]

**Fig. 2.**
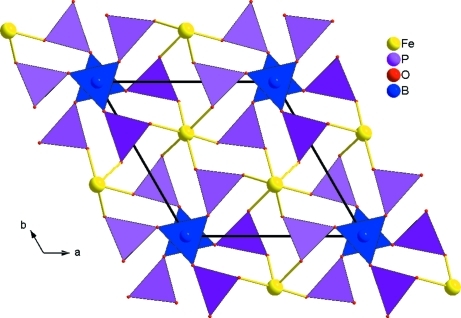
View of the crystal structure of Fe2[BP3012] in a projection along [001].

## supplementary crystallographic information

### Comment

The systematic development of borophosphates has led to a broad spectrum of new borophosphate compounds with quite different anionic partial structures, such as oligomeric units, chains, ribbons, layers, and three-dimensional frameworks. (Kniep *et al.*, 1998; Ewald *et al.*, 2007; Zhao *et al.*, 2009).

Most of the borophosphate compounds were synthesized under hydrothermal conditions; hence, their structures usually incorporate water molecules, hydroxy groups or organic templates. There are considerably less anhydrous borophosphate compounds known, which might have better chemical and thermal stability than the hydrous or templated phases to ensure the feasibility of industrial applications. Herein, we report the redetermined structure of the anhydrous diiron(III) borotriphosphate, Fe_2_[BP_3_O_12_].

The basic building units of the three-dimensional structure of the title compound are FeO_6_ octahedra (3.. symmetry), trigonal-planar BO_3_ groups (6 symmetry) and PO_4_ tetrahedra (*m*.. symmetry) (Fig. 1). Two neighboring FeO_6_ octahedra are connected *via* their faces to form Fe_2_O_9_ dimers. Trigonal-planar BO_3_ units and PO_4_ tetrahedra are isolated. Each BO_3_ triangle connects three PO_4_ tetrahedra *via* corner-sharing O atoms and each PO_4_ connects three Fe_2_O_9_ groups and one BO_3_ group also *via* corner sharing. As shown in Fig. 2, the aforementioned groups are interconnected to form the three-dimensional framework of the title compound. Chen *et al.* (2004) have previously refined the structure of Fe_2_[BP_3_O_12_] in space group *P*3 using the Rietveld method. The analogous chromium compound Cr_2_[BP_3_O_12_] (Mi *et al.*, 2000) is isotypic to this structure model. The differences between the previous and the current model are discussed in the *Refinement Section*. The three-dimensional frameworks of Cr_2_[BP_3_O_12_] (space group *P*3) and our model of Fe_2_[BP_3_O_12_] (space group *P*6_3_/*m*) are very similar, and the differences mainly lie in the distortion of the *M*O_6_ octahedra (*M* = Cr, Fe). The asymmetric unit of Cr_2_[BP_3_O_12_] consists of four Cr atoms, and the Cr–O bond distances range from 1.88 (2) to 2.07 (2) Å, while there is only one Fe site in the asymmetric unit of Fe_2_[BP_3_O_12_] with Fe–O bond distances ranging from 1.929 (2) to 2.103 (2) Å. Based on the current findings, a space group change from *P*3 to *P*6_3_/*m* seems to be most likely for the Cr compound but has to be evidenced experimentally. Meisel *et al.* (2004) have reported the analogous vanadium(III) compound V_2_[BP_3_O_12_] in space group *P*6_3_/*m*, but with a tripled unit cell (*a* of the V compound ≈ 3^1/2^ × *a* of the Fe and Cr compounds). However, a comparison of the three structures shows very similar frameworks.

### Experimental

Single crystals of Fe_2_[BP_3_O_12_] have been prepared by the high temperature solution growth (HTSG) method in air. A powder mixture of Fe_2_O_3_, B_2_O_3_ and NaPO_3_ at the molar ratio of Fe: B: Na: P = 1:5:10:10 was first ground in an agate mortar and then transferred to a platinum crucible. The sample was gradually heated in air at 1173 K for 24 h. In this stage, the reagents were completely melted. After that, the intermediate product was slowly cooled to 673 K at the rate of 2 K h^-1^ and then quenched to room temperature. The obtained crystals were light-red and of prismatic shape. The dimensions of the used sample were typical for the grown crystals in this batch.

### Refinement

Chen *et al.* (2004) have refined the structure of Fe_2_[BP_3_O_12_] using the Rietved method from powder X-ray data and determined the space group to be *P*3, in analogy with the chromium compound Cr_2_[BP_3_0_12_] (Mi *et al.*, 2000). However, in our study we determined the structure from single-crystal X-ray diffraction data in the centrosymmetric space group *P*6_3_/*m*. In the progress of the space group determination using *XPREP* (Sheldrick, 2008), the mean |*E*E*-1| statistics gave a value of 0.948 revealing that the structure is centrosymmetric; the CFOM (combined figure-of-merit) value for each space group determination were *P*3 (16.06), *P*3 (7.16), *P*6_3_ (7.56), *P*6_3_/*m* (1.75). So we selected the latter space group to solve the structure. The  final refinement converged with satisfactory results (*R*_1_(gt) = 0.0348). Furthermore, the final refined model was checked with the ADDSYM algorithm using the program *PLATON* (Spek, 2009), and no higher symmetry was found. Hence, our final structure model is considered to be reasonable and corrects the previous model by Chen *et al.* (2004).

The highest peak in the difference electron density map is located at a distance of 1.41 Å from the Fe1 site while the deepest hole is at a distance of 0.83 Å from the same site.

### Crystal data

**Table:**

Fe_2_[BP_3_O_12_]	*D*_x_ = 3.263 Mg m^−^^3^
*M**_r_* = 407.42	Mo *K*α radiation, λ = 0.71073 Å
Hexagonal, *P*6_3_/*m*	Cell parameters from 1049 reflections
Hall symbol: -P 6c	θ = 4.0–27.4°
*a* = 8.0347 (8) Å	µ = 4.15 mm^−^^1^
*c* = 7.4163 (13) Å	*T* = 293 K
*V* = 414.63 (9) Å^3^	Prism, light-red
*Z* = 2	0.15 × 0.05 × 0.05 mm
*F*(000) = 396	

### Data collection

**Table:**

Rigaku Mercury70 CCD diffractometer	345 independent reflections
Radiation source: fine-focus sealed tube	338 reflections with *I* > 2σ(*I*)
Graphite Monochromator	*R*_int_ = 0.042
Detector resolution: 14.6306 pixels mm^-1^	θ_max_ = 27.4°, θ_min_ = 2.9°
ω scans	*h* = −10→10
Absorption correction: multi-scan (*ABSCOR*; Higashi, 1995)	*k* = −10→10
*T*_min_ = 0.575, *T*_max_ = 0.819	*l* = −9→6
3247 measured reflections	

### Refinement

**Table:**

Refinement on *F*^2^	0 restraints
Least-squares matrix: full	Primary atom site location: structure-invariant direct methods
*R*[*F*^2^ > 2σ(*F*^2^)] = 0.035	Secondary atom site location: difference Fourier map
*wR*(*F*^2^) = 0.072	*w* = 1/[σ^2^(*F*_o_^2^) + (0.0168*P*)^2^ + 3.*P*] where *P* = (*F*_o_^2^ + 2*F*_c_^2^)/3
*S* = 1.07	(Δ/σ)_max_ < 0.001
345 reflections	Δρ_max_ = 0.58 e Å^−^^3^
33 parameters	Δρ_min_ = −0.78 e Å^−^^3^

### Special details

**Table:**

Geometry. All e.s.d.'s (except the e.s.d. in the dihedral angle between two l.s. planes) are estimated using the full covariance matrix. The cell e.s.d.'s are taken into account individually in the estimation of e.s.d.'s in distances, angles and torsion angles; correlations between e.s.d.'s in cell parameters are only used when they are defined by crystal symmetry. An approximate (isotropic) treatment of cell e.s.d.'s is used for estimating e.s.d.'s involving l.s. planes.
Refinement. Refinement of *F*^2^ against ALL reflections. The weighted *R*-factor *wR* and goodness of fit *S* are based on *F*^2^, conventional *R*-factors *R* are based on *F*, with *F* set to zero for negative *F*^2^. The threshold expression of *F*^2^ > σ(*F*^2^) is used only for calculating *R*-factors(gt) *etc*. and is not relevant to the choice of reflections for refinement. *R*-factors based on *F*^2^ are statistically about twice as large as those based on *F*, and *R*- factors based on ALL data will be even larger.

### Fractional atomic coordinates and isotropic or equivalent isotropic displacement parameters (Å^2^)

**Table:**

	*x*	*y*	*z*	*U*_iso_*/*U*_eq_	
Fe1	0.6667	0.3333	0.45190 (12)	0.0072 (3)	
P1	1.04513 (17)	0.68473 (17)	0.2500	0.0064 (3)	
O2	0.8735 (5)	0.4782 (5)	0.2500	0.0080 (7)	
O1	0.9428 (5)	0.8099 (5)	0.2500	0.0097 (8)	
B1	1.0000	1.0000	0.2500	0.0101 (19)	
O3	1.1626 (3)	0.7289 (3)	0.4200 (4)	0.0115 (6)	

### Atomic displacement parameters (Å^2^)

**Table:**

	*U*^11^	*U*^22^	*U*^33^	*U*^12^	*U*^13^	*U*^23^
Fe1	0.0076 (3)	0.0076 (3)	0.0063 (5)	0.00381 (15)	0.000	0.000
P1	0.0055 (6)	0.0057 (6)	0.0077 (7)	0.0025 (5)	0.000	0.000
O2	0.0067 (15)	0.0059 (16)	0.0099 (19)	0.0021 (13)	0.000	0.000
O1	0.0060 (16)	0.0056 (16)	0.017 (2)	0.0022 (13)	0.000	0.000
B1	0.008 (2)	0.008 (2)	0.014 (5)	0.0040 (12)	0.000	0.000
O3	0.0116 (12)	0.0115 (12)	0.0127 (15)	0.0066 (10)	−0.0045 (11)	−0.0019 (10)

### Geometric parameters (Å, °)

**Table:**

Fe1—O3^i^	1.929 (2)	P1—O2	1.538 (3)
Fe1—O3^ii^	1.929 (2)	P1—O1	1.586 (3)
Fe1—O3^iii^	1.929 (2)	O2—Fe1^iv^	2.103 (2)
Fe1—O2^iv^	2.103 (2)	O1—B1	1.357 (3)
Fe1—O2	2.103 (2)	B1—O1^vii^	1.357 (3)
Fe1—O2^v^	2.103 (2)	B1—O1^viii^	1.357 (3)
P1—O3	1.507 (3)	O3—Fe1^i^	1.929 (2)
P1—O3^vi^	1.507 (3)		
			
O3^i^—Fe1—O3^ii^	97.83 (11)	O3—P1—O3^vi^	113.6 (2)
O3^i^—Fe1—O3^iii^	97.83 (11)	O3—P1—O2	111.85 (12)
O3^ii^—Fe1—O3^iii^	97.83 (11)	O3^vi^—P1—O2	111.85 (12)
O3^i^—Fe1—O2^iv^	93.65 (11)	O3—P1—O1	108.24 (12)
O3^ii^—Fe1—O2^iv^	91.53 (11)	O3^vi^—P1—O1	108.24 (12)
O3^iii^—Fe1—O2^iv^	164.04 (11)	O2—P1—O1	102.37 (18)
O3^i^—Fe1—O2	91.53 (11)	P1—O2—Fe1	129.13 (10)
O3^ii^—Fe1—O2	164.04 (11)	P1—O2—Fe1^iv^	129.14 (10)
O3^iii^—Fe1—O2	93.65 (11)	Fe1—O2—Fe1^iv^	90.78 (13)
O2^iv^—Fe1—O2	74.92 (10)	B1—O1—P1	136.3 (3)
O3^i^—Fe1—O2^v^	164.04 (11)	O1^vii^—B1—O1^viii^	120.000 (1)
O3^ii^—Fe1—O2^v^	93.65 (11)	O1^vii^—B1—O1	120.000 (1)
O3^iii^—Fe1—O2^v^	91.53 (11)	O1^viii^—B1—O1	120.000 (1)
O2^iv^—Fe1—O2^v^	74.92 (10)	P1—O3—Fe1^i^	142.54 (17)
O2—Fe1—O2^v^	74.92 (10)		

Symmetry codes: (i) −*x*+2, −*y*+1, −*z*+1; (ii) *x*−*y*, *x*−1, −*z*+1; (iii) *y*, −*x*+*y*+1, −*z*+1; (iv) −*x*+*y*+1, −*x*+1, −*z*+1/2; (v) −*y*+1, *x*−*y*, *z*; (vi) *x*, *y*, −*z*+1/2; (vii) −*y*+2, *x*−*y*+1, *z*; (viii) −*x*+*y*+1, −*x*+2, −*z*+1/2.
